# Supplementary material for: Public economic gains from tax-financed investments in childhood immunization in the United States
Source: PLOS Glob Public Health. 2023 Oct 18;3(10):e0002461. doi: 10.1371/journal.pgph.0002461 (PMC10584131; doi:10.1371/journal.pgph.0002461)
Supplement: S1 Table — (DOCX) [file pgph.0002461.s001.docx]

**S1 Table Clinical study reporting outcomes linking to fiscal consequences**

| **Study** | **Data inputs used in the model** |
| --- | --- |
| Winsor et al., 2019 | - Reduction of earnings for individuals with any disability: 29.38%   calculated as [(35.1–49.7)/49.7] x 100   - Reduction of earnings for individuals with cognitive disability: 50.91%   calculated as [(24.4–49.7)/49.7] x 100 |
| Emmett and Francis, 2015 | - Reduction of economic activity/employment for individuals with hearing loss: 50.5%   calculated as (1/1.98) |
| Newman et al., 2011 | - Reduction of earnings for individuals with hearing loss: 7.89% calculated as [(10.5–11.4)/11.4] x 100 |
| Note: Winsor et al. (2019) report a comprehensive overview of the U.S. national trends in employment for people with intellectual and developmental disabilities for all 50 states and the District of Columbia . The reported reductions in earnings for individuals with any disability or cognitive disability were 29.38% and 50.91%, respectively. Employment and wage reductions were applied to the annual incomes of the general U.S. population . Emmett and Francis (2015) report associations between hearing loss and educational attainment, income, and unemployment/underemployment in U.S. adults. This study included adult (aged 20–69 years) participants in the 1999–2002 cycles of the National Health and Nutrition Examination Survey. The study reports data on audiometric evaluations and income levels, educational attainment, and unemployment or underemployment. The impact of hearing loss on earnings comes from the National Longitudinal Transition Study-2. The study reports data from a nationally representative sample of high school students with disabilities over a 10-year period. Reductions of earnings for individuals with hearing loss are reported to be 7.89%.  Sources  Winsor J, Timmons J, Butterworth J, Migliore A, Domin D, Zalewska A, et al. StateData: The National Report on Employment Services and Outcomes Through 2017. Institute for Community Inclusion (UCEDD), University of Massachusetts Boston, 2019.  Emmett SD, Francis HW. The socioeconomic impact of hearing loss in U.S. adults. Otol Neurotol. 2015;36(3):545-50. doi: 10.1097/MAO.0000000000000562. PubMed PMID: 25158616; PubMed Central PMCID: PMCPMC4466103.  Newman L, Wagner M, Knokey A-M, Marder C, Nagle K, Shaver D, et al. The Post-High School Outcomes of Young Adults With Disabilities up to 8 Years After High School: A Report From the National Longitudinal Transition Study-2 (NLTS2). U.S. DEPARTMENT OF EDUCATION, 2011. | |
