# Supplementary material for: Public economic gains from tax-financed investments in childhood immunization in the United States
Source: PLOS Glob Public Health. 2023 Oct 18;3(10):e0002461. doi: 10.1371/journal.pgph.0002461 (PMC10584131; doi:10.1371/journal.pgph.0002461)
Supplement: S2 Table — (DOCX) [file pgph.0002461.s002.docx]

**S2 Table U.S. Census Bureau median and mean income from Current Population Survey**

| **Age group** | **Median income (US$)** | **Mean income (US$)** |
| --- | --- | --- |
| 15–24 | $13,344 | $19,874 |
| 25–29 | $35,552 | $43,004 |
| 30–34 | $42,341 | $54,170 |
| 35–39 | $49,269 | $66,034 |
| 40–44 | $48,867 | $67,247 |
| 45–49 | $49,990 | $72,510 |
| 50–54 | $50,798 | $71,243 |
| 55–59 | $46,025 | $69,276 |
| 60–64 | $39,252 | $61,243 |
| 65–69 | $32,711 | $53,516 |
| 70–74 | $29,011 | $48,307 |
| 75+ | $23,581 | $38,592 |
| Source: U.S. Census Bureau, Current Population Survey, 2020 Annual Social and Economic Supplement.  Note: The analysis linked to age-specific earnings quantifies lifetime transfers to the government in the form of direct and indirect taxes. The age-specific income for all workers collected by the U.S. Census Bureau was used to calculate human capital losses from vaccine-preventable infectious diseases in children for mortality and disability. Earnings were inflated to reflect real wage growth over the duration of working years and adjusted for labor force participation rate by age obtained from the U.S. Bureau of Labor Statistics. | | |
