# Supplementary material for: Public economic gains from tax-financed investments in childhood immunization in the United States
Source: PLOS Glob Public Health. 2023 Oct 18;3(10):e0002461. doi: 10.1371/journal.pgph.0002461 (PMC10584131; doi:10.1371/journal.pgph.0002461)
Supplement: S3 Table — (DOCX) [file pgph.0002461.s003.docx]

**S3 Table Labor force participation**

| **Age group** | **Labor force participation (%)** |
| --- | --- |
| 16–19 | 28.5% |
| 20–24 | 69.0% |
| 25–34 | 81.9% |
| 35–44 | 82.2% |
| 45–54 | 81.3% |
| 55–64 | 68.7% |
| 65–74 | 33.2% |
| 75–80 | 11.8% |
| Source: U.S. Bureau of Labor Statistics. Civilian labor force participation rates by age, sex, race, and ethnicity, projected 2029 (in percent). | |
