# Supplementary material for: Public economic gains from tax-financed investments in childhood immunization in the United States
Source: PLOS Glob Public Health. 2023 Oct 18;3(10):e0002461. doi: 10.1371/journal.pgph.0002461 (PMC10584131; doi:10.1371/journal.pgph.0002461)
Supplement: S4 Table — (DOCX) [file pgph.0002461.s004.docx]

**S4 Table Nonrelated healthcare (Medicaid) costs**

| **Age group** | **Annual Medicaid cost (US$) 2014** | **Out-of-pocket expenses (US$) 2014** | **Annual Medicaid costs (US$) adjusted 2019, excludes patient costs^†^** | **Weighting** |
| --- | --- | --- | --- | --- |
| 0–18 | $3,367 | $382 | $3,991 | 38% |
| 19–44 | $4,285 | $571 | $5,093 | 15% |
| 45–64 | $8,976 | $1,236 | $10,675 | 15% |
| Source: NHSG. Centers for Medicare and Medicaid Services, Office of the Actuary. In: Group NHS, editor. 2019.  Note: The proportion of people likely to receive Medicaid varies by age with 38% of those ages 0–18 receiving support and 15% of the adult population (19–64) Source: Bureau UC. Medicaid State Fact Sheets. In: KFF KFF, editor. State Health Facts. Washington2020. The annual costs per Medicaid beneficiary were weighted using the proportions of people receiving Medicaid over the ages of 1–65. At the age of 65 we consider that all individuals will transfer to Medicare.  ([Annual per capita cost 2014] – [Annual OOP cost 2014]) x CPI = Annual cost per Medicaid recipient (US$) adjusted 2019 | | | | |
