# Supplementary material for: Public economic gains from tax-financed investments in childhood immunization in the United States
Source: PLOS Glob Public Health. 2023 Oct 18;3(10):e0002461. doi: 10.1371/journal.pgph.0002461 (PMC10584131; doi:10.1371/journal.pgph.0002461)
Supplement: S1 Text — (DOCX) [file pgph.0002461.s005.docx]

**S1 Text Disability costs and special education costs**

In the United States, parents of children with physical and mental challenges and adults with disabilities that prevent them from achieving a typical working life receive disability support. The duration of adolescent disability payments was included up to age 21. In the United States, the disability payment in adulthood is not measured in terms of actual physical limitations, rather, the reduced earning capacity of affected people is considered. In this regard, people with disabilities are encouraged to work as adults, and the government seeks to supplement income accordingly. In 2019, the average social security disability insurance payment for all disabled workers was $1,234 per month. The disability payment made to parents of children with disabilities was, on average, $686 per month .

Cognitive disability results in special education costs borne by states. These costs are captured as fiscal transfer costs in the pediatric vaccines fiscal model. Previous studies have reported that the costs of educating children with disabilities are 2.2-fold higher than the costs of educating children without disabilities . Similarly, a comparable 2.5-fold expenditure increase was reported in New York. This 2.2 factor was applied to the national spending average of $12,612 per pupil reported by the U.S. Census Bureau for children experiencing cognitive disability during schooling years defined as ages 5–20.

Sources:

Administration SS. The Faces and Facts of Disability. In: Administration SS, editor.: Social Security Administration; 2020.

Petek G. Overview of Special Education in California. California.

NYSED. Fiscal Accountability Summary (2016-17). New York State Government; 2017.

Bureau UC. U.S. School System Spending Per Pupil by Region. 2020 11 May 2020. Report No.
