# Supplementary material for: Public economic gains from tax-financed investments in childhood immunization in the United States
Source: PLOS Glob Public Health. 2023 Oct 18;3(10):e0002461. doi: 10.1371/journal.pgph.0002461 (PMC10584131; doi:10.1371/journal.pgph.0002461)
Supplement: S2 Text — (DOCX) [file pgph.0002461.s006.docx]

**S2 Text Costs of longevity paid by government**

The analysis considers the additional fiscal costs attributable to changes in survival patterns from vaccination, described as longevity costs in the analysis. Longevity costs include unrelated age-specific Medicare and Medicaid costs and Social Security payments, and costs of those children with permanent disabilities dependent on social security. To reflect these costs, we apply the reported estimates of the proportions of individuals receiving Medicaid at different stages of life. The per capita cost for those receiving Medicaid was based on annual expenditure per person reported in 2014 and adjusted to 2019 based on inflation estimates provided by the Health Resources and Services Administration . From the age-specific cost per person we deducted the reported out-of-pocket (OOP) expenditure participants paid to reflect the net cost. The annual cost per Medicare enrollee applied in the model was $10,536 (2019).

The average per person Medicare costs after reaching age 65 are also included . Individuals reaching the retirement age of 65 are eligible for Social Security benefits. The average monthly benefit of $1,514 reported by the Social Security Administration was applied in the model .

Sources:

HRSA. Consumer Price Index (CPI) for Medical Care 2020 [cited 2021 20 March 2021]. Available from: https://www.hrsa.gov/sites/default/files/hrsa/get-health-care/affordable/hill-burton/cpitables.pdf.

Foundation KF. Medicare Spending Per Enrollee. kff.org: Kaiser Family Foundation, 2021.

Security S. Fact Sheet Social Security. In: Administration SS, editor. Washington DC2020. p. 2.
