# Supplementary material for: Public economic gains from tax-financed investments in childhood immunization in the United States
Source: PLOS Glob Public Health. 2023 Oct 18;3(10):e0002461. doi: 10.1371/journal.pgph.0002461 (PMC10584131; doi:10.1371/journal.pgph.0002461)
Supplement: S3 Text — (DOCX) [file pgph.0002461.s007.docx]

**S3 Text Estimation of lifetime direct and indirect taxes**

Taxes paid comprise three components: payroll taxes, federal income tax, and state/local taxes. The percentage of annual earnings paid to each state by citizens was obtained from the Tax Foundation Facts & Figures Annual Report for 2020 . The Tax Foundation collects the percentage of earnings of individuals that transfer to state and local governments in the form of sales taxes, state income taxes, property taxes, and various levies applied at state and local levels. To reflect the national average, we applied the reported average for the United States of 9.9% to age-specific earnings . The contribution of earnings to payroll taxes for Social Security and Medicare by the employer and employee of 7.65% each were applied to age-specific earnings every year . Income tax rates are progressive in the United States with tax brackets between 10% and 37%. The reported average income tax by the Tax Foundation is 14.9% of gross earnings. This value was applied to annual age-specific earnings in the model.

Source:

Foundation T. Facts & Figures, How Does Your State Compare? Washington DC: Tax Foundation, 2020 ISBN 978-1-942768-24-1.

Foundation T. The US Tax Burden on Labor, 2019. Washington DC: Tax Foundation, 2019 Contract No.: No. 656.
