# Supplementary material for: Public economic gains from tax-financed investments in childhood immunization in the United States
Source: PLOS Glob Public Health. 2023 Oct 18;3(10):e0002461. doi: 10.1371/journal.pgph.0002461 (PMC10584131; doi:10.1371/journal.pgph.0002461)
Supplement: S4 Text — (DOCX) [file pgph.0002461.s008.docx]

**S4 Text Cost inflation, discounting, and wage growth**

In this analysis, we consider all real (inflation-adjusted) government costs or benefits made available to individuals to be tax-financed transfer payments. Benefits were discounted at a rate of 3% . Government transfer payments were inflated according to the Consumer Price Index (CPI) by 1.2% . This approach is consistent with public economic assessments conducted by government to reflect real costs . Annual real wage growth was set at 3.1% .

Sources:

Sanders GD, Neumann PJ, Basu A, Brock DW, Feeny D, Krahn M, et al. Recommendations for conduct, methodological practices, and reporting of cost-effectiveness analyses: second panel on cost-effectiveness in health and medicine. Jama. 2016;316(10):1093-103.

Statistics BL. Consumer Price Index. In: Statistics BoL, editor. Washington DC: US Bureau of Labor Statistics 2020.

Auerbach AJ, Gokhale J, Kotlikoff LJ. Generational accounting: a meaningful way to evaluate fiscal policy. Journal of Economic Perspectives. 1994;8(1):73-94.

Real Earnings - November 2020 [Internet]. Bureau of Labor Statistics; 2020; 10 December 2020.
