# Supplementary material for: Public economic gains from tax-financed investments in childhood immunization in the United States
Source: PLOS Glob Public Health. 2023 Oct 18;3(10):e0002461. doi: 10.1371/journal.pgph.0002461 (PMC10584131; doi:10.1371/journal.pgph.0002461)
Supplement: Attachment — Submitted filename: Response to Reviewers PLOS Global Health.docx [file pgph.0002461.s009.docx]

Reviewer #1: REVIEW

PGPH-D-23-00704

Public economic gains from tax-financed investments in childhood immunization in the United States
Thank you for asking me to review the paper. The paper estimates the monetary benefits of deaths and disabilities averted from vaccine that prevents several illnesses that can occur anytime from a very early age (although some only during childhood). It calculates lifetime wages from workers who are statistically alive or not disabled due to the vaccine interventions. It also calculates net contributions to the US fiscal balance: taxes minus longevity entitlements and disability transfers. It compares the present value of the estimate to the vaccine costs. This is a very worthwhile exercise and has been done accurately using currently available data. The paper deserves to be published. This exercise is worth carrying out and should be presented to Congress and other governance bodies.

Response: We appreciate your feedback and acknowledgement of the importance of our work. We agree that this type of data can be used by government bodies to consider ongoing investments in state sponsored vaccination programs.

I don’t have much to say; I will make only a few points.

1. It would be nice to get some basic numbers with an example. This would give the general picture of your assumptions and some cogency to the numbers. Diphtheria, for example, has a case-fatality rate of 5-10% but could be 20% for anyone < 5 years of age and > 40 years of age. How did you pick the number of years lived when a death is avoided. What are the assumptions of the base wage for example. This is just to get a handle on this for the reader when they read it. Some of this is in the supplementary material.

Response: Thanks for raising these important points in relation to our analysis.

When a death is avoided, we assumed that the individual would follow the death rates from the life tables for the United States. We did not assume any residual mortality effect from the prior exposure for all conditions, therefore irrespective of the prior infection all individuals died at the annual mortality rate defined by the life tables. We have added some information regarding this point to the revised manuscript.

The wages are based on age-specific means reported by the US Census Bureau where the information on wages has been reported in the Supplemental material. Because wages are an important component of this analysis, we have added a description to the Methods section to provide some details for the readers regarding wages. The tabulated wage data is included in the Supplement.

2. My own exercise at the median wage estimated that a person’s tax contribution to social security and Medicare transfer has a ratio around 2.4 (PV $190,000 tax to $-90,000 transfer). This roughly is about 41% amount going to longevity transfer; I am assuming cost of education and other things are covered from the $190k. The amount 41% might be slightly an overestimate from reality; but not too far off. But averting a death amounts to a net gain of $100,000 present value. What is the corresponding value for averting a disability? An estimate: Suppose a person becomes disabled at the age of 21 and lives 30 years more and government pays net transfer of $50000 per year for care. The care has a present value of $550,000 as costs. Suppose the question is to avert a death or a disability at the margin. Disability always wins out for the government. This is not to say the approach taken here is at all wrong. It would be nice to simply to say that this methodology at this time is intended only to show that vaccine programs with most plausible assumptions are likely to yield a great deal of benefit. However, making decisions on human lives based on net fiscal benefits is not a good idea. It is just to note some limitations.

Response: The results reported in our analysis apply a cohort approach in which disabled, non-disabled and deaths are included with the results reported in aggregate. The model applies a wage penalty for disability and for death from which the government loses tax revenue. What you state is true in that the government gains more from averting a disability due to the disability costs, however in our reported we have opted to report the findings in aggregate rather than disaggregated by disability status or death.

As you have highlighted an important concept, we have highlighted this point in the Discussion of our revised manuscript.

Reviewer #2: The paper studies the value of childhood vaccinations from the perspective of the US government. The paper is interesting and written well. It is widely acknowledged, at least in academic circles, that vaccination provides substantial benefits. The rationale the paper provides for studying the value is “to inform debate over regular negotiations regarding public vaccination funding.” To articulate the decision(s) the analysis supports (and how it can be used), it would be useful to provide more background on these negotiations and the debate beyond estimating the value of individual vaccines and comparing their value to decide which should be included in the vaccination programme.

Response: The framework for negotiations between the government and manufacturers of vaccines is described by government legislation. In the revised manuscript we have provided a reference for how these negotiations are performed. In practice, the details of individual negotiations and prices are not publicly available, hence it is difficult to provide more depth on this important topic.

The analysis is thorough and conducted well.

My primary query is about the longevity costs. The unrelated future costs are comparatively low. How do the unrelated future costs of vaccines relative to related costs compare to other studies? In this study, are they just accounted for from >64 yo? I do expect costs to be highest later in life but also suspect these are heavily discounted at this point, being quite far into the future. And, with vaccine-preventable deaths occurring early in life, I think that would have explained the much lower longevity costs. However, scenarios 3 & 4 in Table 2, seem to go in the opposite direction: not discounting yields greater benefits without longevity effect and lower benefits with longevity effect. This suggests that discounting is not the reason. Could it be due to the shorter timeframes over which later-life diseases can the overall value?

Response: The longevity costs over the age of 65 include healthcare costs and social security payments i.e., pensions. Prior to age 65 we also include the unrelated health costs for those people receiving healthcare through tax-finance health systems i.e., Medicaid in the US. This is briefly described in the Methods Lines 205 – 208 with additional detail provided in the Supplemental material. We have provided additional information in the revised manuscript and direct people to the Supplement for further details.

This is a good observation. The results from scenario 4 are not discounted. As we are inflating future government costs e.g., pensions and healthcare costs, this results in a very low fiscal BCR of 1.6 which is the opposite of what one might expect. This result is driven by the inflation rate over the long-time horizon. The modeling approach is consistent with generational accounting methodology and the longevity costs are inflated as per CPI.

I was also wondering how representative the unrelated future costs are for the cohort of newborns in both scenarios – with and without vaccines. Additionally, how do the methods for unrelated future costs compare to methods in the literature on estimating this cost – e.g., van Baal et al. (2011) in Pharmacoeconomics?

Response: In our analysis we include the lifetime unrelated healthcare costs for those receiving tax funded healthcare through Medicaid and Medicare. As we are applying a strictly fiscal perspective, we do not account for private insurance coverage and those costs paid out of pocket by individuals. The per person cost data used in our analysis is reported by the Centers for Medicare and Medicaid Services in the United States. This data has been included in the Supplemental material and we reproduce in the table below for clarity.

**Table S5	Nonrelated healthcare (Medicaid) costs**

| **Age group** | **Annual Medicaid cost (US$) 2014** | **Out-of-pocket expenses (US$) 2014** | **Annual Medicaid costs (US$) adjusted 2019, excludes patient costs^†^** | **Weighting** |
| --- | --- | --- | --- | --- |
| 0–18 | $3,367 | $382 | $3,991 | 38% |
| 19–44 | $4,285 | $571 | $5,093 | 15% |
| 45–64 | $8,976 | $1,236 | $10,675 | 15% |
| Source: Centers for Medicare and Medicaid Services, Office of the Actuary, National Health Statistics Group | | | | |

The major difference between our approach and that of van Baal is that we have to adjust for the proportion of people on state funded Medicaid under the age of 65. In contrast, most people in the Netherlands are receiving healthcare through the social health insurance scheme. As shown in the table below we applied a weighting for those on Medicaid below age 65 to determine the “per person” costs for unrelated healthcare costs.

For those over the age of 65, we apply the average cost per person by age reported by Medicare.

6. PLOS authors have the option to publish the peer review history of their article ([what does this mean?](https://journals.plos.org/globalpublichealth/s/editorial-and-peer-review-process#loc-peer-review-history)). If published, this will include your full peer review and any attached files.


**Do you want your identity to be public for this peer review?** If you choose “no”, your identity will remain anonymous but your review may still be made public.


For information about this choice, including consent withdrawal, please see our [Privacy Policy](https://www.plos.org/privacy-policy).

Reviewer #1: **Yes:** Arnab K Acharya, PhD, MPH

Reviewer #2: No
